# Anticancer effects of the PLK4 inhibitors CFI-400945 and centrinone in Ewing’s sarcoma cells

**DOI:** 10.1007/s00432-020-03346-z

**Published:** 2020-08-08

**Authors:** Sophie L. Kerschner-Morales, Marie Kühne, Sabine Becker, James F. Beck, Jürgen Sonnemann

**Affiliations:** 1 Department of Paediatric Haematology and Oncology, Children’s Clinic, Jena University Hospital, Jena, Germany; 2 Research Centre Lobeda, Jena University Hospital, Jena, Germany; 3 CMB, Institute for Biochemistry and Biophysics, Friedrich Schiller University, Jena, Germany; 4 Klinik für Kinder- Und Jugendmedizin, Friedrich-Schiller-Universität Jena, Am Klinikum 1, 07747 Jena, Germany

**Keywords:** Ewing’s sarcoma, Polo-like kinase 4, CFI-400945, Centrinone, Apoptosis, Cell cycle

## Abstract

**Purpose:**

Polo-like kinase 4 (PLK4) inhibitors, such as CFI-400945 and centrinone, are emerging as promising antineoplastic agents. However, their effectiveness against Ewing’s sarcoma, a highly aggressive childhood cancer, remains to be established.

**Methods:**

CFI-400945 and centrinone were tested in three Ewing’s sarcoma cell lines with different *TP53* status. Effects were assessed by flow-cytometric analyses of cell death, dissipation of the mitochondrial transmembrane potential and cell cycle distribution, by cell viability assay as well as by caspase 3/7 activity measurement, by immunoblotting and by immunofluorescence microscopy.

**Results:**

CFI-400945 and centrinone elicited cell death in p53 wild-type and mutant Ewing’s sarcoma cells. Both agents induced mitochondrial membrane depolarisation, caspase 3/7 activation, PARP1 cleavage and DNA fragmentation, indicating an apoptotic form of cell death. In addition, the PLK4 inhibitors induced a G2/M cell cycle arrest, particularly when cell killing was attenuated by the pan-caspase inhibitor z-VAD-fmk. Moreover, CFI-400945 treatment produced polyploidy.

**Conclusion:**

Our findings show that PLK4 inhibitors were effective against Ewing’s sarcoma cells in vitro and thus provide a rationale for their evaluation in vivo.

**Electronic supplementary material:**

The online version of this article (10.1007/s00432-020-03346-z) contains supplementary material, which is available to authorized users.

## Introduction

Ewing’s sarcoma (ES) is the second most common bone cancer in children and adolescents (Balamuth and Womer 2010; Grünewald et al. 2018). The pathognomonic feature of ES is the gene fusion of a member of the *FET* gene family (consisting of *FUS*, *EWSR1* and *TAF15*), by far most frequently *EWSR1* (*EWS RNA binding protein 1*), with a member of the *ETS* gene family of transcription factors, most commonly *FLI1*. The resulting fusion protein is causative for neoplastic transformation and tumour progression; other mutations at diagnosis are rare (Grünewald et al. 2018). ES is one of the most aggressive childhood cancers: patients with localised disease have a survival probability of about 75%, while of those with detectable metastasis at initial presentation less than 40% survive (Balamuth and Womer 2010; Grünewald et al. 2018). The standard of care is a treatment combination consisting of cytotoxic chemotherapy, surgery and radiation (Balamuth and Womer 2010). Of note, the prognosis for patients with ES has reached a plateau over the last two decades, as no further therapy improvement by optimising treatment protocols could be achieved (Casey et al. 2019). It is thus imperative to identify druggable targets to improve the outcome for ES patients.

This has spurred us to conduct a systematic exploration of potentially clinically actionable targets. So far, special emphasis was placed on the family of histone deacetylases (HDACs), i.e., on the inhibition of class I and II HDACs (Sonnemann et al. 2007) as well as on the modulation of the sirtuin class of HDACs (Marx et al. 2018; Sonnemann et al. 2016). Other efforts aimed at drugging the p53 system (Sonnemann et al. 2011, 2015). We have recently extended this exploration to polo-like kinases (PLKs), a family of five serine/threonine protein kinases, whose dysfunction is associated with cancer (Archambault et al. 2015). PLKs play a key role in cell cycle control and progression (Zitouni et al. 2014). Their structure contains a C-terminal polo-box (PB) domain consisting of two or three PBs which mediate substrate interaction. In addition, PLKs contain an amino-terminal kinase domain responsible for substrate phosphorylation (Zitouni et al. 2014).

To date, most attention on PLKs as anticancer targets has focused on PLK1 (Lee et al. 2015), yet presently also PLK4 is emerging as an opportunity for inferring antineoplastic strategies (Maniswami et al. 2018; Zhao and Wang 2019). PLK4 is essential for centriole biogenesis and plays an important role in the mediation of cytokinesis (Habedanck et al. 2005; Press et al. 2019). It differs in its structure from the other PLK family members in that it contains a cryptic PB domain with two PBs and an additional third single PB (Zitouni et al. 2014). The cryptic PB enables the homodimerization of PLK4, in turn leading to autophosphorylation and activation of its catalytic function. Active PLK4 is responsible for the initiation and control of centriole duplication (Zitouni et al. 2014). PLK4 is also involved in the regulation of cell motility and migration (Zhao and Wang 2019) and its overexpression can promote metastasis (Kazazian et al. 2017). PLK4 has been found to be aberrantly expressed in patient-derived tumour samples, further underscoring its potential as a therapeutic target (Zhao and Wang 2019).

A few PLK4-targeting small molecule compounds have been developed (Maniswami et al. 2018; Zhao and Wang 2019). A systematic drug discovery programme using breast cancer cells led to the identification of the PLK4 inhibitor (PLK4i) CFI-400945 (Mason et al. 2014; Sampson et al. 2015). CFI-400945 is an ATP-competitive inhibitor with a *K*_*i*_ of 0.26 nM and an IC_50_ of 2.8 nM. It is selective for PLK4 over PLK1-3, but inhibits aurora B kinase with an IC_50_ of 98 nM (Mason et al. 2014). CFI-400945 is orally active, and it is currently undergoing clinical trials in patients with diverse cancers (Zhao and Wang 2019). Other PLK4i are the structurally and functionally closely related centrinone and centrinone-B, which reversibly inhibit PLK4 with a Ki of 0.16 nM and 0.6 nM, respectively, and show > 1000-fold selectivity for PLK4 over aurora kinases (Wong et al. 2015). Centrinone-B was effective against melanoma cells in a preclinical study (Denu et al. 2018).

All told, the targeting of PLK4 appears to be a promising new anticancer strategy. As to childhood cancers, PLK4 has been reported to be overexpressed in patient-derived rhabdoid tumour and neuroblastoma samples (Sredni et al. 2017b; Tian et al. 2018; Bailey et al. 2018). Moreover, PLK4i have been shown to exert anticancer activities against cultured rhabdoid tumour, medulloblastoma and neuroblastoma cells (Sredni et al. 2017a, b; Suri et al. 2019; Tian et al. 2018), but they have not yet been tested in ES cells. Therefore, we examined the PLK4i CFI-400945 and centrinone in ES cell lines in vitro, and we found them to be effective in inducing cell death and cell cycle arrest.

## Material and methods

### Cell culture

WE-68 cells were a gift from Dr F. van Valen (Münster, Germany). SK-ES-1 and HeLa cells were purchased from the DSMZ (Braunschweig, Germany). A673 cells were purchased from Sigma Aldrich (Deisenhofen, Germany). WE-68, SK-ES-1 and HeLa cells were cultured in RPMI 1640 medium and A673 cells were cultured in DMEM (Lonza, Cologne, Germany). Media were supplemented with 10% foetal calf serum (Capricorn Scientific, Ebsdorfergrund, Germany), 2 mM l-glutamine, 100 units/ml penicillin G sodium and 100 µg/ml streptomycin sulphate (Lonza). All tissue culture vessels used for the cultivation of ES cells were coated with rat tail collagen (Merck, Darmstadt, Germany) at a concentration of 5 µg/cm^2^. Cells were maintained at a temperature of 37 °C in a humidified 5% CO_2_ incubator and routinely passaged at a confluence of ~ 90%. Cells were tested to be negative for mycoplasma with the qPCR Mycoplasma Test Kit from Applichem (Darmstadt, Germany).

### Treatment of cells

For flow-cytometric, caspase 3/7 activity and PCR analyses, WE-68 and SK-ES-1 cells were seeded in 12-well tissue culture plates and A673 cells were seeded in 6-well tissue culture plates. For flow-cytometric and PCR analyses, WE-68 and SK-ES-1 cells were seeded at a density of 150,000 cells/well, and A673 cells were seeded at a density of 100,000 cells/well. For measurement of caspase 3/7 activity, all cells were seeded at a density of 200,000 cells/well. For cell viability assays, cells were seeded in 96-well tissue culture plates; WE-68 and SK-ES-1 cells were seeded at a density of 3000 (72 h incubation) or 4000 (48 h incubation) cells/well, A673 cells were seeded at a density of 2000 (72 h incubation) or 3000 (48 h incubation) cells/well. Cells were treated with centrinone (0.5–3 µM; MedChem Express, Monmouth Junction, NJ, USA) or CFI-400945 (10–50 nM; MedChem Express) for 12–72 h, depending on the read-out. In the respective experiments, cells were pre-exposed to 20 µM z-VAD-fmk (Enzo Life Sciences, Lörrach, Germany) 1 h before treatment with PLK4i. In the combination experiments, cells were coexposed to PLK4i and etoposide (provided by the Jena University Hospital Pharmacy) for 48 h and 72 h.

### Real-time RT-PCR

Total RNA was isolated using the Peqgold Total RNA Kit including DNase digestion (Peqlab, Erlangen, Germany). RNA was transcribed into cDNA using Omniscript (Qiagen, Hilden, Germany). Real-time PCR for *PLK4* was performed using the Thermo Fisher Scientific (Dreieich, Germany) Applied Biosystems 7900HT Real-Time PCR system. *PLK4* expression levels were normalised to *B2M* expression levels. Reactions were done in duplicate using Applied Biosystems Gene Expression Assays (*PLK4*: Hs00179514_m1, *B2M*: Hs00187842_m1) and Universal PCR Master Mix. All procedures were performed according to the manufacturers’ protocols. The relative gene expressions were calculated by the 2^(−ΔΔCt)^ method.

### Flow-cytometric analysis of cell death

Cell death was measured by determining the integrity of the cell membrane by flow-cytometric analysis of propidium iodide (PI; Sigma Aldrich) uptake. Cells were incubated in 2 µg/ml PI in PBS for 5 min at 4 °C in the dark. 10,000 cells per sample were analysed on a BD (Heidelberg, Germany) FACSCanto II using BD FACSDiva software; data were gated to exclude debris.

### Cell viability assay

The assays were done in quadruplicates in 96-well plates. At the end of the treatment period, 1/10 volume of the resazurin solution (Promocell, Heidelberg, Germany) was added and cells were incubated at 37 °C for additional 3 h. The fluorescent signal of reduced resazurin was measured on a Tecan (Crailsheim, Germany) Infinite M200 Pro plate reader using an excitation/emission wave length of 560/590 nM. Results are expressed as the ratio of fluorescence of treated to untreated cells.

### Flow-cytometric analysis of mitochondrial transmembrane potential (Δ*ψ*_m_) decay

The loss of Δ*ψ*_*m*_ was measured using 3,3′-dihexyloxacarbocyanine iodide [DiOC_6_(3)] (Thermo Fisher Scientific). Before harvesting, cells were incubated with 50 nM DiOC_6_(3) for 45 min at 37 °C. 10,000 cells per sample were analysed on a BD FACSCanto II; data were gated to exclude debris.

### Flow-cytometric analysis of DNA content

DNA content was measured according to Riccardi and Nicoletti (2006). After harvesting, cells were washed with PBS and fixed in 70% ice-cold ethanol over night at − 20 °C. After washing with PBS, cells were incubated in 500 µl DNA extraction buffer consisting of 200 mM Na_2_HPO_4_ and 0.1% Triton X-100 (pH 7.8) for 5 min at room temperature. Cells were washed in PBS, resuspended in PBS containing 20 µg/ml PI and 200 µg/ml RNase A (Roche, Mannheim, Germany) and incubated for 30 min at room temperature in the dark. 20,000 cells per sample were analysed on a BD FACSCanto II. The different cell cycle phases were quantified using FACSDiva software; data were gated to exclude debris.

### Caspase 3/7 activity

Caspase 3/7 activity was assessed by measuring the fluorogenic substrate Ac-DEVD-AMC (Bachem, Weil am Rhein, Germany). After harvesting, cells were lysed in 10 mM Tris–HCl, 10 mM NaH_2_PO_4_/NaHPO_4_ (pH 7.5), 130 mM NaCl, 1% Triton X-100 and 10 mM Na_4_P_2_O_7_ for 15 min at 4 °C in the dark. The samples were mixed with activation buffer consisting of 20 mM Hepes (pH 7.5), 10% glycerol, 2 mM DTT and 25 µg/ml Ac-DEVD-AMC and incubated for 2 h at 37 °C. The release of AMC was measured with a BMG Labtech (Offenburg, Germany) FLUOstar Omega or a Tecan Infinite M200 Pro using excitation/emission wavelengths of 355/460 nM. The relative caspase 3/7 activities were calculated as a ratio of the emission of treated to untreated cells.

### Immunoblotting

3 × 10^6^ (WE-68, SK-ES-1) or 1.2 × 10^6^ cells (A673) were seeded in petri dishes and harvested after a 2-h or 24-h treatment, washed with PBS and lysed in RIPA buffer [50 mM Tris/HCl (pH 8.0), 150 mM NaCl, 1 mM EDTA, 1% Triton X-100, 1% sodium deoxycholate and 0.1% SDS] supplemented with protease and phosphatase inhibitor cocktails (Roche) for 30 min at 4 °C, followed by sonication for 3 min and centrifugation at 4 °C for 15 min to separate lysate from debris. After SDS–polyacrylamide gel electrophoresis, proteins were transferred to PVDF membrane (Carl Roth, Karlsruhe, Germany) using NuPAGE Transfer Buffer (Thermo Fisher Scientific). Membranes were blocked for 1 h with 5% dry milk in PBS at room temperature, followed by incubation with antibodies over night at 4 °C. Primary antibodies used were rabbit anti-PARP1 (Cell Signaling, Danvers, MA, USA, #9542; 1:1000), mouse anti-vinculin (Bio-Rad, Feldkirchen, Germany, #MCA465GA; 1:10.000), mouse anti-p53 (Santa Cruz Biotechnology, Heidelberg, Germany, #sc-126; 1:2000) and mouse anti-phospho-histone H2A.X Ser139 (Merck, #05–636; 1:1000). HRP-conjugated goat anti-rabbit IgG (Thermo Fisher Scientific, #31,460; 1:2000) or HRP-conjugated goat anti-mouse IgG (Thermo Fisher Scientific, #31,430; 1:2000) was used as secondary antibodies followed by detection of specific signals using Supersignal West Pico Plus Chemiluminescent Substrate (Thermo Fisher Scientific). Imaging was carried out using a Fusion Solo S imager (Vilber Lourmat, Eberhardzell, Germany).

### Immunofluorescence microscopy

WE-68 cells were seeded on collagen-coated cover slips. 24 h after treatment, cells were fixed in 1 ml methanol for 5 min at − 20 °C and washed three times in PBS. Prior to staining, each sample was blocked in 1% BSA in PBS for 20 min at room temperature. Cells were incubated with a rat anti-α-tubulin antibody (Bio-Rad, #MCA77G; 1:500) for 1 h at room temperature. Cover slips were washed three times with PBS, followed by incubation with a secondary Cy3-conjugated goat anti-rat antibody (Jackson ImmunoResearch, Ely, UK, #112–165-167; 1:1000) for 45 min at room temperature in the dark. Finally, cover slips were washed three times with PBS and stained with 5 µg/ml DAPI (Sigma) for 20 min at room temperature, followed by repeated washing with PBS and sealing with Fluorescence Mounting Medium (Agilent, Waldbronn, Germany). Mounted cells were imaged using a Zeiss (Jena, Germany) Axiovert 200 microscope.

### Statistical analysis

Statistical significance of differences between experimental groups was evaluated by the paired two-tailed Student’s *t* test.

## Results

### Antineoplastic effects of CFI-400945 and centrinone in ES cells

**Fig. 1:**
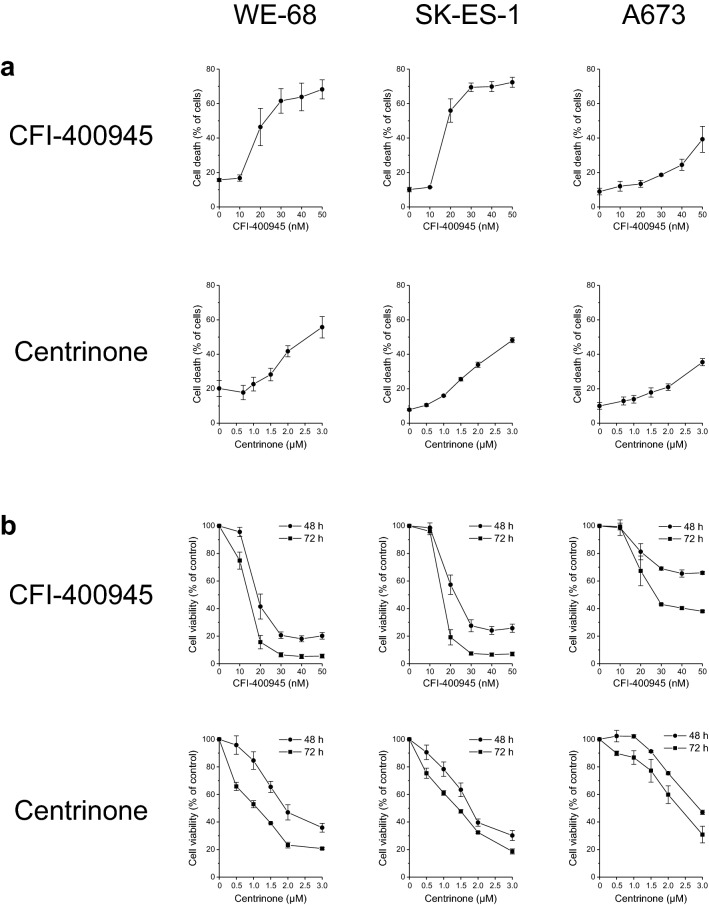
Antineoplastic effects of PLK4i in ES cells. Cells were exposed to CFI-400945 or centrinone for 48 h (PI uptake analysis, cell viability assay) and 72 h (cell viability assay). **a** Cell death was determined by flow-cytometric analysis of PI uptake. **b** Cell viability was determined by resazurin assay. Means ± SEM of each three separate measurements are shown

Of the compounds that have been developed to inhibit PLK4 (Maniswami et al. 2018; Zhao and Wang 2019), we selected CFI-400945 and centrinone for investigation in cultured ES cells. CFI-400945 is the only PLK4i that has been tested in a clinical trial (Veitch et al. 2019), and centrinone has been found to be the most selective PLK4i in a study exploring several agents for their PLK4-inhibitory action (Suri et al. 2019). Since a study on melanoma cells observed that centrinone-B was less effective in cells with mutant p53 (Denu et al. 2018), we used three ES cell lines with different *TP53* status, i.e., wild-type p53 WE-68 cells, mutant p53 (C176F) SK-ES-1 cells (Sonnemann et al. 2015) and p53 null A673 cells (Ottaviano et al. 2010), to address a potential impact of p53 on the response of ES cells to PLK4 inhibition. Initially, we determined the relative gene expression levels of *PLK4* in the three cell lines by real-time RT-PCR. These measurements revealed that *PLK4* expression levels were considerably higher in ES cells than in HeLa cells (Fig. S1), thus underscoring the potential of PLK4 as drug target in ES. To examine the antineoplastic effects of CFI-400945 and centrinone in ES cells, we first measured cell death by flow-cytometric analysis of PI uptake. Figure 1a shows that the PLK4i induced cell death in a concentration-dependent manner in the three cell lines. CFI-400945 was active at nanomolar concentrations, while centrinone was effective in the low micromolar range, in accordance with their different *K*_*i*_ values (Mason et al. 2014; Wong et al. 2015). WE-68 and SK-ES-1 cells reacted similarly, albeit slightly more sensitively than A673 cells to the treatment, suggesting that PLK4i-mediated cell killing in ES cells did not hinge on functional p53. We also assessed the effects of CFI-400945 and centrinone by resazurin-based cell viability assay. As presented in Fig. 1b, the results of these assays reflect those of the PI uptake analyses: CFI-400945 and centrinone affected cell viability in a concentration- and time-dependent fashion and were somewhat more effective in WE-68 and SK-ES-1 cells than in A673 cells.

It has been reported that polyploidy can render cancer cells more susceptible to DNA-damaging agents (Hau et al. 2006). Consistently, CFI-400945 has been shown to enhance the cytotoxic activity of the genotoxic drugs etoposide and doxorubicin in rhabdoid tumour and medulloblastoma cells (Sredni et al. 2017a). We thus tested whether CFI-400945 and centrinone could increase the susceptibility of ES cells to etoposide, a standard drug for the treatment of ES (Anderton et al. 2020), by cell viability assay. As shown in Fig. S2, the effect of combined treatment was not more than additve in ES cells.

**Fig. 2:**
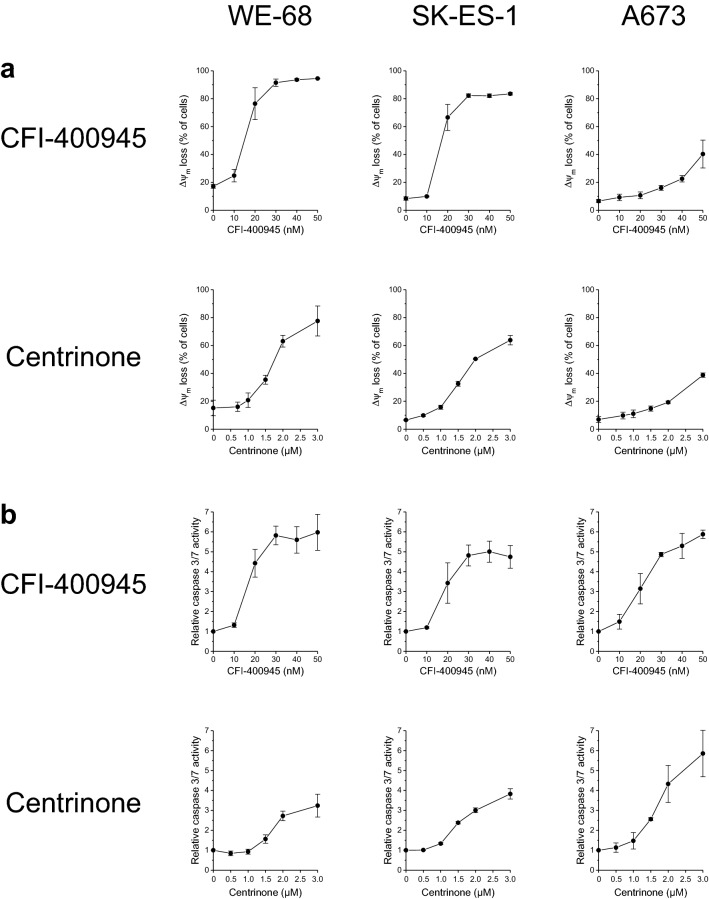

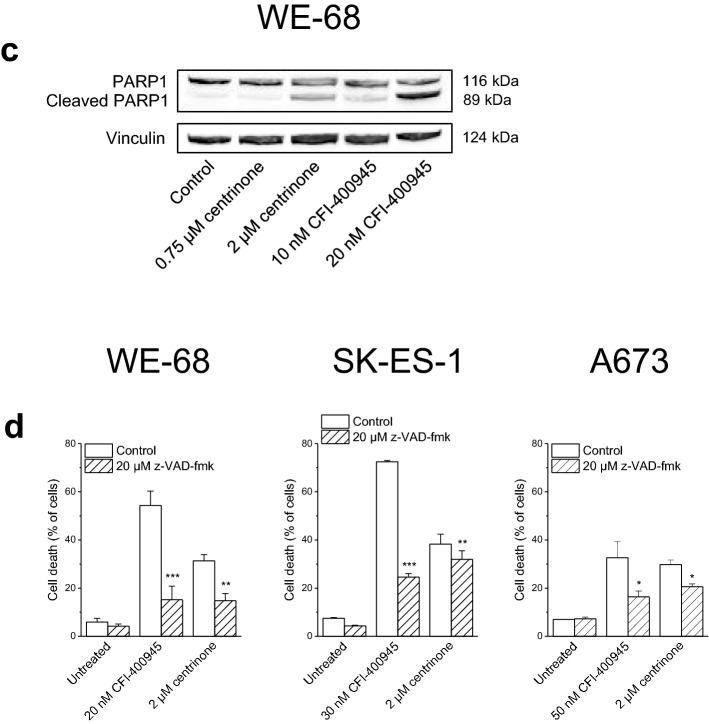
Induction of cell death in ES cells by PLK4i involves apoptosis. Cells were exposed to CFI-400945 or centrinone for 24 h (**b**, **c**) or 48 h (**a**, **d**). **a** Δ*ψ*_*m*_ loss was determined by flow-cytometric analysis of DiOC_6_(3) staining. **b** Caspase 3/7 activity was determined using the fluorogenic substrate Ac-DEVD-AMC; relative caspase 3/7 activities are the ratio of treated cells to untreated cells. **c** PARP1 cleavage was determined by immunoblotting. **d** Cell death was determined by flow-cytometric analysis of PI uptake; z-VAD-fmk was applied 1 h before treatment with PLK4i. **a**, **b**, **d** Means ± SEM of each three separate measurements are shown (*P < 0.05, **P < 0.01, ***P < 0.001). **c** The figure is representative of three independent determinations

To evaluate if cell death caused by CFI-400945 and centrinone involved apoptosis, we examined Δ*ψ*_*m*_ loss, caspase 3/7 activity, PARP1 cleavage and the effect of the pan-caspase inhibitor z-VAD-fmk on PLK4i-elicited cell death. Consistent with the cell death results, the PLK4i triggered a concentration-dependent decay of Δ*ψ*_*m*_, with a weaker response again being observed in A673 cells (Fig. 2a). Caspase 3/7 activity measurements revealed that both agents activated caspase 3/7 in the three cell lines (Fig. 2b). As an additional marker for apoptotic cell death, we determined PARP1 processing in WE-68 cells by immunoblotting. Figure 2c depicts that CFI-400945 and centrinone treatment provoked the appearance of the 89-kDa PARP1 cleavage product in a concentration-dependent manner. To test whether caspase 3/7 activation was not a mere side action but essential for PLK4i-induced cell death, we applied z-VAD-fmk in the PI uptake analyses. Figure 2d shows that the pan-caspase inhibitor decreased cell killing caused by CFI-400945 and centrinone.

### Cell cycle effects of CFI-400945 and centrinone in ES cells

**Fig. 3:**
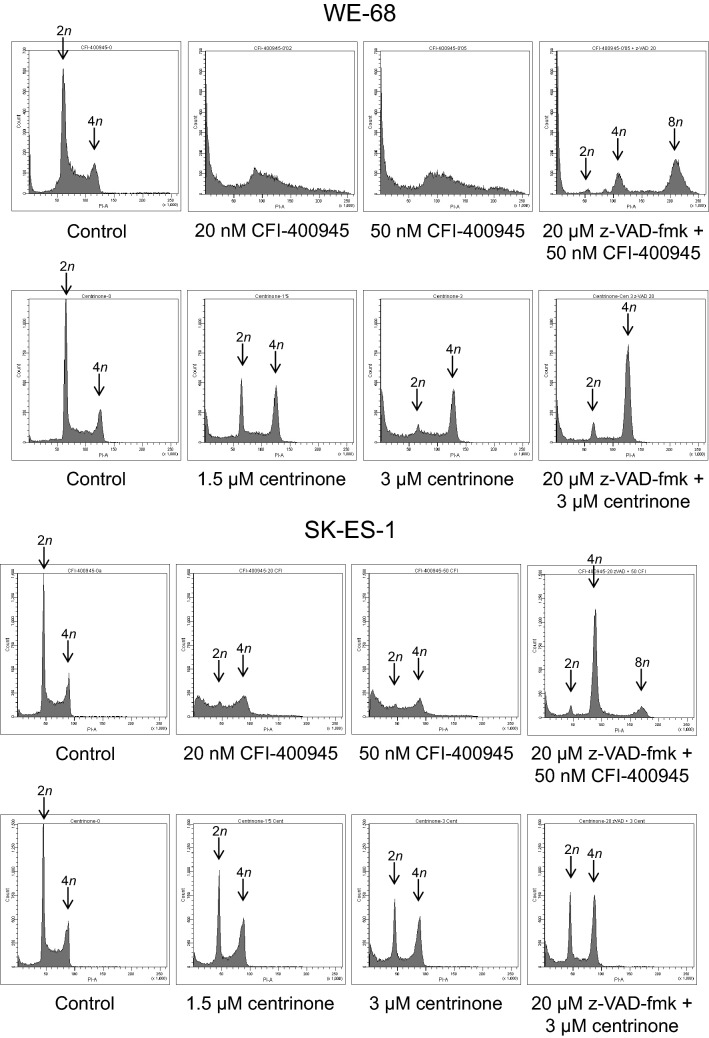

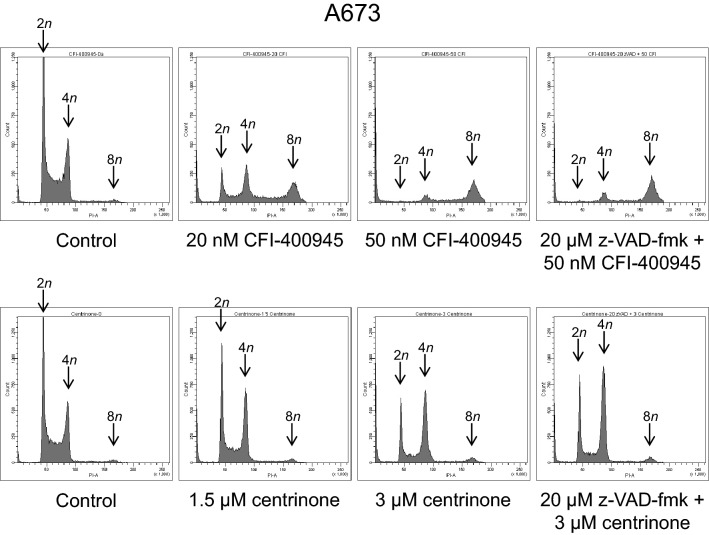
PLK4i induce cell cycle effects in ES cells. Cells were exposed to CFI-400945 or centrinone for 48 h; z-VAD-fmk was applied 1 h before treatment with PLK4i. Cell cycle profiles were determined by flow-cytometric analysis of PI-stained ethanol-fixed cells. The figure is representative of each three independent measurements

As PLK4 is substantially involved in cell cycle processes, we examined effects of the PLK4i on the cell cycle of ES cells by staining the DNA of ethanol-fixed cells with PI, followed by determining the DNA content by flow cytometry. After a 48-h treatment, both inhibitors elicited an increase of cells with fragmented DNA < 2*n* in a concentration-dependent fashion (Fig. 3; the full data set is shown in Tables S1 to S6), further substantiating the apoptosis-inducing action of CFI-400945 and centrinone. However, the PLK4i differed in their effects on cell cycle progression in the three cell lines. Treatment with centrinone resulted in the accumulation of cells in the G2/M phase, i.e., in a G2/M arrest, while treatment with CFI-400945 predominantly promoted the emergence of polyploid cells (8*n*). Both these effects were even more evident when apoptosis was blocked by z-VAD-fmk.

**Fig. 4:**
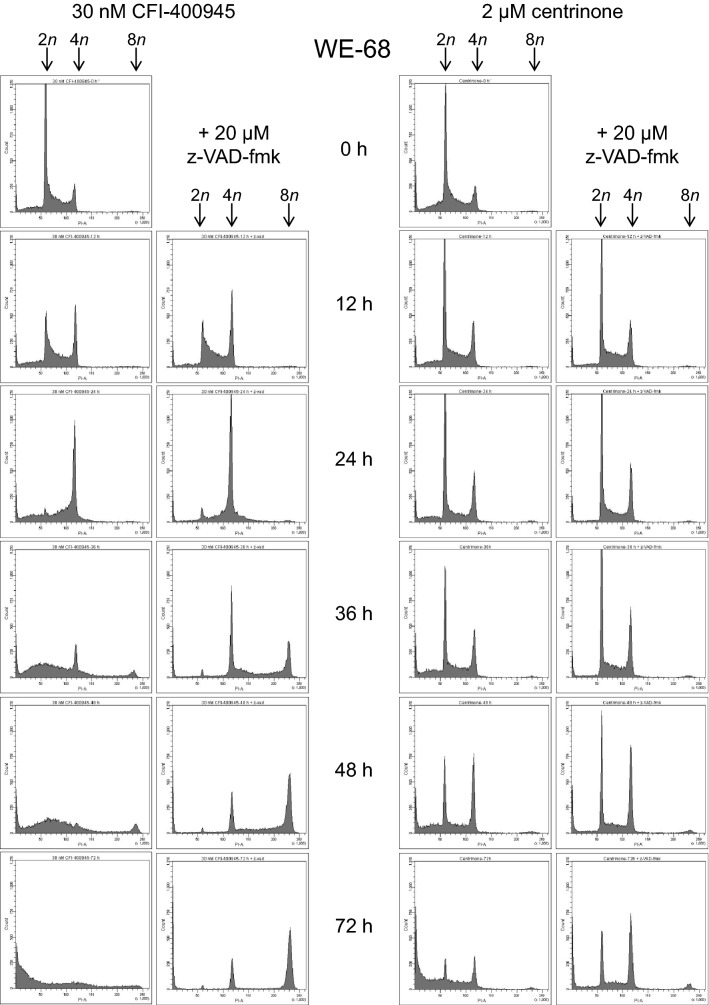
Time course of PLK4i-induced cell cycle effects in WE-68 cells. Cells were exposed to 30 nM CFI-400945 or 2 µM centrinone for the indicated times; z-VAD-fmk was applied 1 h before treatment with PLK4i. Cell cycle profiles were determined by flow-cytometric analysis of PI-stained ethanol-fixed cells. The figure is representative of three independent measurements

To gain a more in-depth understanding of the cell cycle responses to PLK4i treatment, we conducted time course analyses over 72 h with fixed concentrations of CFI-400945 (30 nM) and centrinone (2 µM) in WE-68 cells. These analyses most notably revealed a more detailed picture of CFI-400945′s cell cycle effects: a CFI-400945-induced G2/M cell cycle arrest became visible after shorter treatment periods. The G2/M arrest unfolded after 12 h, peaked after 24 h and returned to baseline after 48 h (Fig. 4; the full data set is shown in Tables S7 and S8). Hence, the cell cycle arrest preceded the induction of apoptosis and the appearance of polyploid cells which set in after 36 h. Also in these analyses, G2/M arrest and polyploidy became especially manifest when caspase activities were repressed by z-VAD-fmk.

To complement these data, we assessed WE-68 cells by immunofluorescence microscopy using an α-tubulin antibody. In keeping with the results from the cell cycle analyses, CFI-400945 treatment led to the appearance of multinucleated cells and the formation of multipolar spindles (Fig. S3a). These effects became more noticeable upon coincubation with z-VAD-fmk (Fig. S3b).

### Effects of CFI-400945 and centrinone on DNA damage

**Fig. 5:**
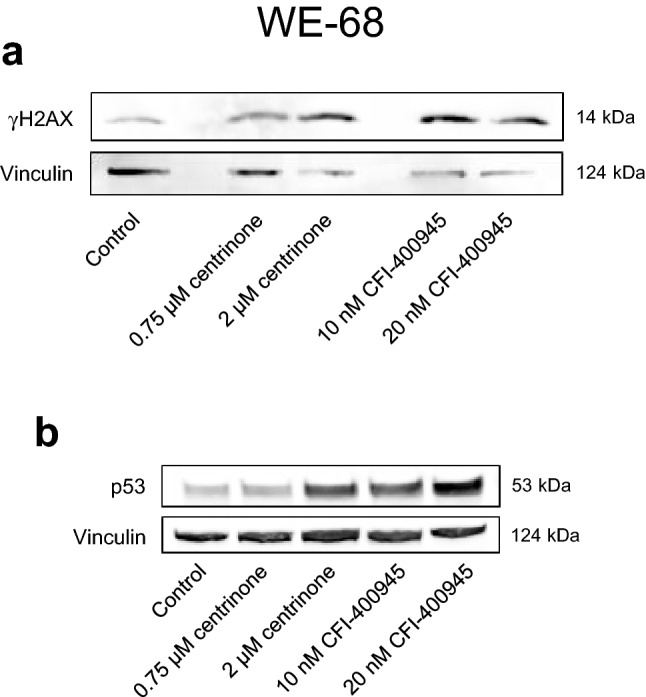
PLK4i induce DNA damage in WE-68 cells. Cells were exposed to CFI-400945 or centrinone for 2 h (**a**) or 24 h (**b**). **a** H2AX phosphorylation and **b** p53 expression levels were determined by immunoblotting. The figures are representative of each three independent determinations. The loading control in **b** is the same as in Fig. 2c since PARP1, p53 and vinculin were detected on the same blots

As the standard chemotherapy of ES rests on DNA-damaging drugs, such as ifosfamide, doxorubicin and etoposide (Anderton et al. 2020), which entail a considerable risk of causing secondary malignancies (Marina et al. 2017), the application of less genotoxic agents is desirable. We therefore examined whether the PLK4i caused DNA damage by determining the phosphorylation of histone H2AX (γH2AX) by immunoblotting. Figure 5a shows that CFI-400945 and centrinone induced H2AX phosphorylation in WE-68 cells. In line with the occurrence of γH2AX, we also observed an increase in the abundance of p53, another typical feature of the DNA damage response (Williams and Schumacher 2016) (Fig. 5b). PLK4i thus do not spare DNA damage.

## Discussion

ES is one of the most malignant childhood cancers and its prognosis has not improved over the past two decades (Casey et al. 2019; Vornicova and Bar-Sela 2016). There thus is an urgent need for the development of new therapeutic approaches (Bailey et al. 2019). Recent studies point to PLK4 as a promising antineoplastic target (Maniswami et al. 2018; Zhao and Wang 2019). The PLK4i CFI-400945 and centrinone have shown anticancer activity in several tumours including a few paediatric malignancies (Sredni et al. 2017a, b; Suri et al. 2019; Tian et al. 2018), but they have not yet been investigated in ES. Our study demonstrates that CFI-400945 and centrinone were effective against ES cells.

Although cell death can proceed via several pathways, the mitochondrial pathway of apoptosis is viewed as most relevant to cancer treatment, for it is typically harnessed by anticancer agents (Bhola and Letai 2016). Likewise, the PLK4i have been reported to trigger cell death by inducing apoptosis (Denu et al. 2018; Kawakami et al. 2018a). Several lines of evidence emerging from our study indicate that they invoked cell death through mitochondrial apoptosis also in ES cells. We found that exposure to CFI-400945 or centrinone led to Δ*ψm* dissipation, caspase 3/7 activation and DNA fragmentation, three common characteristics of apoptosis. Induction of apoptosis was additionally confirmed by detection of PLK4i-mediated PARP1 cleavage. Most importantly, experiments using the pan-caspase inhibitor z-VAD-fmk revealed that active caspases were essential for PLK4i-elicited cell death. z-VAD-fmk impinged slightly more effectively on CFI-400945-induced than on centrinone-induced cell death, pointing to a somewhat greater role of caspases in CFI-400945-elicited cell killing.

The results from our examination of PLK4i-mediated cell death in ES cells suggest that CFI-400945 and centrinone act in a largely similar way. However, certain differences between the compounds emerged from the cell cycle analyses. When cell cycle effects were determined at a fixed time point (at 48 h; see Fig. 3 and Tables S1 to S6), CFI-400945 treatment predominantly produced polyploid cells, while centrinone treatment resulted in a G2/M cell cycle arrest. Different effects of CFI-400945 and centrinone have already been observed in a lung cancer cell line, leading to the conclusion that CFI-400945′s effects were not solely due to PLK4 inhibition (Oegema et al. 2018). An alternative explanation for the dissimilar effects, however, has been put forward in support of a PLK4-selective activity of CFI-400945 (Kawakami et al. 2018b). Our findings do not provide a conclusive answer to this debate, but they indicate that differences between the two PLK4i could be in part due to the chosen measurement time: when the cell cycle was monitored at 12-h intervals, both agents were found to induce a G2/M cell cycle arrest, albeit with different kinetics (see Fig. 4). It should also be noted that CFI-400945 and centrinone inhibit PLK4 with considerably different dissociation constants (Mason et al. 2014; Wong et al. 2015), necessitating different dosages of the compounds and, consequently, complicating their comparability. CFI-400945 has been described to exert a concentration-dependent bimodal effect: low concentrations led to centriole overduplication, whereas high concentrations blocked centriole duplication (Kawakami et al. 2018a; Mason et al. 2014). However, as a general note on the specificity of protein kinase inhibitors, it may be taken into account that these agents are rarely fully selective. A comprehensive study on kinase inhibitor selectivity of 243 clinically tested kinase drugs revealed that the vast majority of the inhibitors interfered with kinases in addition to the one intended (CFI-400945 and centrinone were not included in the study) (Klaeger et al. 2017).

In any case, the response of ES cells to PLK4i treatment was dominated by apoptosis induction, especially after longer treatment periods. When cells were protected from apoptosis by the pan-caspase inhibitor z-VAD-fmk, however, CFI-400945 and centrinone treatment produced a pronounced cell cycle effect. This finding implies that the PLK4i were capable of eliciting growth arrest when their apoptosis-inducing activity was blocked. Disabled apoptosis can reduce the efficacy of chemotherapy (Holohan et al. 2013). CFI-400945 and centrinone thus may be useful for the treatment of ES with curtailed apoptotic responsiveness due to defects in the caspase system.

Even more importantly, we found that CFI-400945 and centrinone exerted anticancer activity in ES cells with different *TP53* status, indicating that their action was independent of functional p53. This finding is in contrast to results obtained in melanoma cells, in which centrinone-B was found to be less effective in cells with mutant *TP53* (Denu et al. 2018), yet in line with p53-independent effects of CFI-400945 observed in lung cancer cells (Kawakami et al. 2018a). These dissimilar results point to a context-dependent role of p53 in the response to PLK4 inhibition. In any case, the evidently p53-independent action of CFI-400945 and centrinone in ES cells is a welcome finding from the clinical perspective: *TP53* mutations are relatively rare in ES though (Grünewald et al. 2018), the subset of patients with mutant *TP53* has a considerably poorer outcome than average (Crompton et al. 2014; Tirode et al. 2014). It is therefore of clinical relevance that the PLK4i were effective against ES cells irrespective of their *TP53* status.

Our results presented here establish the potential of CFI-400945 and centrinone for the treatment of ES, although further studies are certainly required to confirm that these findings hold up in vivo. It should also not be overlooked that PLK4i exposure resulted in DNA damage, raising the concern that these agents may entail the long-term sequelae typical of genotoxic drugs. Nonetheless, the potent anticancer effects of CFI-400945 and centrinone—which did neither spare p53 mutant nor apoptotically impaired ES cells—render them a promising new option for the treatment of ES.

## Electronic supplementary material

Below is the link to the electronic supplementary material.

Supplementary file1 (DOCX 1467 kb)

## Data Availability

All data sets on which the conclusions of the manuscript rely are presented in the paper and its supplementary information files.
