# Supplementary material for: Anticancer effects of the PLK4 inhibitors CFI-400945 and centrinone in Ewing’s sarcoma cells
Source: J Cancer Res Clin Oncol. 2020 Aug 8;146(11):2871–83. doi: 10.1007/s00432-020-03346-z (PMC7519924; doi:10.1007/s00432-020-03346-z)
Supplement: Supplementary file 1 — Supplementary file1 (DOCX 1467 kb) [file 432_2020_3346_MOESM1_ESM.docx]

**Anticancer effects of the PLK4 inhibitors CFI-400945 and centrinone in Ewing's sarcoma cells**

Sophie L. Kerschner-Morales, Marie Kühne, Sabine Becker, James F. Beck, Jürgen Sonnemann

**Supplementary Tables**

**Table S1. CFI-400945 induces cell cycle effects in WE-68 cells.**

| CFI-400945 | control | 10 nM | 20 nM | | 30 nM | | 40 nM | | 50 nM | |
| --- | --- | --- | --- | --- | --- | --- | --- | --- | --- | --- |
| –/+ z-VAD-fmk | – | – | – | + | – | + | – | + | – | + |
| <2*n* (% of cells) | 7.0 ± 0.47 | 12.2 ± 5.09 | 25.0 ± 2.27 | 16.2 ± 3.71 | 25.6 ± 0.69 | 16.5 ± 1.84 | 26.5 ± 1.82 | 16.8 ± 2.11 | 26.3 ± 0.78 | 18.9 ± 0.87 |
| 2*n* (% of cells) | 44.7 ± 0.98 | 42.6 ± 2.20 | 11.2 ± 6.60 | 7.7 ± 3.29 | 6.0 ± 1.60 | 2.5 ± 0.64 | 6.0 ± 0.96 | 2.5 ± 0.47 | 5.9 ± 0.89 | 2.4 ± 0.49 |
| *>*2*n*–*<*4*n* (% of cells) | 24.8 ± 0.51 | 22.7 ± 2.11 | 21.4 ± 0.78 | 13.8 ± 3.89 | 19.7 ± 1.16 | 4.6 ± 0.31 | 20.0 ± 2.36 | 5.2 ± 0.71 | 19.3 ± 0.51 | 5.0 ± 0.62 |
| 4*n* (% of cells) | 17.7 ± 1.11 | 16.4 ± 1.09 | 14.0 ± 0.24 | 18.4 4.09 | 13.5 ± 0.84 | 15.9 ± 1.29 | 13.4 ± 0.49 | 15.1 ± 0.98 | 13.3 ± 0.40 | 14.3 ± 1.27 |
| *>*4*n* (% of cells) | 5.9 ± 0.73 | 6.1 ± 1.00 | 28.5 ± 4.80 | 43.9 ± 5.84 | 35.1 ± 2.84 | 60.5 ± 2.33 | 34.1 ± 3.89 | 60.4 ± 2.96 | 35.4 ± 0.76 | 59.5 ± 1.60 |

Cells were exposed to CFI-900495 for 48 h; 20 µM z-VAD-fmk was applied 1 h before treatment with CFI-900495. The percentage of cells in each cell cycle phase was determined by flow-cytometric analysis of PI-stained ethanol-fixed cells. Means ± SEM of three separate measurements are shown.

**Table S2. Centrinone induces cell cycle effects in WE-68 cells.**

| Centrinone | control | 0.5 µM | 1 µM | 1,5 µM | 2 µM | | 3 µM | |
| --- | --- | --- | --- | --- | --- | --- | --- | --- |
| –/+ z-VAD-fmk | – | – | – | – | – | + | – | + |
| <2*n* (% of cells) | 4.2 ± 0.43 | 6.1 ± 0.62 | 7.9 ± 0.29 | 17.3 ± 1.58 | 29.5 ± 2.00 | 17.2 ± 0.56 | 28.7 ± 2.93 | 17.7 ± 4.49 |
| 2*n* (% of cells) | 45.7 ± 3.44 | 40.2 ± 3.00 | 38.0 ± 2.64 | 33.6 ± 3.04 | 25.4 ± 4.69 | 20.7 ± 2.40 | 21.4 ± 2.96 | 12.6 ± 2.31 |
| *>*2*n*–*<*4*n* (% of cells) | 21.0 ± 0.73 | 18.7 ± 0.38 | 17.2 ± 0.31 | 16.3 ± 1.53 | 15.5 ± 0.82 | 11.2 ± 1.04 | 16.3 ± 0.78 | 9.5 ± 0.87 |
| 4*n* (% of cells) | 21.0 ± 3.00 | 27.9 ± 2.58 | 29.8 ± 2.47 | 26.1 ± 2.84 | 23.1 ± 3.09 | 38.4 ± 1.07 | 28.4 ± 4.40 | 48.7 ± 5.51 |
| *>*4*n* (% of cells) | 8.2 ± 1.58 | 7.2 ± 1.38 | 7.1 ± 1.36 | 6.8 ± 0.87 | 6.5 ± 0.60 | 12.5 ± 1.80 | 5.2 ± 0.64 | 11.6 ± 1.36 |

Cells were exposed to centrinone for 48 h; 20 µM z-VAD-fmk was applied 1 h before treatment with centrinone. The percentage of cells in each cell cycle phase was determined by flow-cytometric analysis of PI-stained ethanol-fixed cells. Means ± SEM of three separate measurements are shown.

**Table S3. CFI-400945 induces cell cycle effects in SK-ES-1 cells.**

| CFI-400945 | control | 20 nM | | 50 nM | |
| --- | --- | --- | --- | --- | --- |
| –/+ z-VAD-fmk | – | – | + | – | + |
| <2*n* (% of cells) | 7.7 ± 1.62 | 29.5 ± 4.09 | 16.0 ± 6.89 | 38.6 ± 2.13 | 18.3 ± 10.04 |
| 2*n* (% of cells) | 44.0 ± 2.24 | 15.4 ± 5.02 | 10.0 ± 4.00 | 11.0 ± 1.33 | 5.3 ± 0.40 |
| *>*2*n*–*<*4*n* (% of cells) | 22.1 ± 0.62 | 17.6 ± 1.47 | 8.4 ± 3.00 | 14.8 ± 1.09 | 4.5 ± 0.22 |
| 4*n* (% of cells) | 26.0 ± 2.09 | 30.9 ± 2.67 | 47.6 ± 4.82 | 28.0 ± 3.49 | 51.2 ± 7.00 |
| *>*4*n* (% of cells) | 0.3 ± 0.16 | 6.6 ± 1.20 | 18.1 ± 2.27 | 7.6 ± 0.71 | 20.7 ± 2.58 |

Cells were exposed to CFI-900495 for 48 h; 20 µM z-VAD-fmk was applied 1 h before treatment with CFI-900495. The percentage of cells in each cell cycle phase was determined by flow-cytometric analysis of PI-stained ethanol-fixed cells. Means ± SEM of three separate measurements are shown.

**Table S4. Centrinone induces cell cycle effects in SK-ES-1 cells.**

| Centrinone | control | 1.5 µM | | 3 µM | |
| --- | --- | --- | --- | --- | --- |
| –/+ z-VAD-fmk | – | – | + | – | + |
| <2*n* (% of cells) | 7.6 ± 1.98 | 13.5 ± 3.58 | 11.3 ± 2.82 | 24.1 ± 6.20 | 18.2 ± 5.62 |
| 2*n* (% of cells) | 42.9 ± 3.91 | 32.0 ± 2.29 | 34.8 ± 2.18 | 21.8 ± 4.82 | 21.8 ± 4.18 |
| *>*2*n*–*<*4*n* (% of cells) | 22.1 ± 0.82 | 16.5 ± 2.09 | 14.1 ± 0.60 | 10.5 ± 2.18 | 8.9 ± 1.18 |
| 4*n* (% of cells) | 27.0 ± 1.29 | 37.0 ± 0.31 | 38.8 ± 2.33 | 42.4 ± 3.27 | 49.7 ± 4.98 |
| *>*4*n* (% of cells) | 0.4 ± 0.07 | 0.8 ± 0.24 | 1.1 ± 0.24 | 1.2 ± 0.13 | 1.5 ± 0.38 |

Cells were exposed to centrinone for 48 h; 20 µM z-VAD-fmk was applied 1 h before treatment with centrinone. The percentage of cells in each cell cycle phase was determined by flow-cytometric analysis of PI-stained ethanol-fixed cells. Means ± SEM of three separate measurements are shown.

**Table S5. CFI-400945 induces cell cycle effects in A673 cells.**

| CFI-400945 | control | 20 nM | | 50 nM | |
| --- | --- | --- | --- | --- | --- |
| –/+ z-VAD-fmk | – | – | + | – | + |
| <2*n* (% of cells) | 2.1 ± 0.20 | 8.7 ± 1.67 | 4.0 ± 0.42 | 26.6 ± 0.76 | 19.4 ± 0.80 |
| 2*n* (% of cells) | 36.3 ± 1.09 | 14.5 ± 3.07 | 19.1 ± 2.76 | 2.0 ± 0.07 | 2.1 ± 0.18 |
| *>*2*n*–*<*4*n* (% of cells) | 24.4 ± 0.42 | 11.4 ± 2.27 | 14.4 ± 1.60 | 2.5 ± 0.11 | 3.0 ± 0.16 |
| 4*n* (% of cells) | 30.8 ± 0.84 | 26.8 ± 1.31 | 30.1 0.89 | 10.5 ± 0.04 | 14.1 ± 0.53 |
| *>*4*n* (% of cells) | 6.3 ± 0.47 | 38.6 ± 4.93 | 32.5 ± 4.53 | 58.4 ± 0.80 | 61.5 ± 0.76 |

Cells were exposed to CFI-900495 for 48 h; 20 µM z-VAD-fmk was applied 1 h before treatment with CFI-900495. The percentage of cells in each cell cycle phase was determined by flow-cytometric analysis of PI-stained ethanol-fixed cells. Means ± SEM of three separate measurements are shown.

**Table S6. Centrinone induces cell cycle effects in A673 cells.**

| Centrinone | control | 1.5 µM | | 3 µM | |
| --- | --- | --- | --- | --- | --- |
| –/+ z-VAD-fmk | – | – | + | – | + |
| <2*n* (% of cells) | 2.3 ± 0.29 | 7.3 ± 0.27 | 5.4 ± 0.62 | 16.3 ± 2.02 | 10.9 ± 1.49 |
| 2*n* (% of cells) | 39.2 ± 0.20 | 29.2 ± 1.96 | 31.3 ± 1.84 | 16.4 ± 1.67 | 18.7 ± 2.16 |
| *>*2*n*–*<*4*n* (% of cells) | 24.0 ± 0.62 | 16.8 ± 0.40 | 17.2 ± 0.53 | 9.8 ± 0.36 | 10.4 ± 0.73 |
| 4*n* (% of cells) | 29.2 ± 0.18 | 36.9 ± 1.09 | 36.9 ± 1.11 | 47.0 ± 1.64 | 50.5 ± 1.07 |
| *>*4*n* (% of cells) | 5.3 ± 0.58 | 9.8 ± 1.04 | 9.1 ± 0.62 | 10.5 ± 0.20 | 9.5 ± 0.36 |

Cells were exposed to centrinone for 48 h; 20 µM z-VAD-fmk was applied 1 h before treatment with centrinone. The percentage of cells in each cell cycle phase was determined by flow-cytometric analysis of PI-stained ethanol-fixed cells. Means ± SEM of three separate measurements are shown.

**Table S7. Time course of CFI-400945-induced cell cycle effects in WE-68 cells.**

| Incubation time | 0 h | 12 h | | 24 h | | 36 h | | 48 h | | 72 h | |
| --- | --- | --- | --- | --- | --- | --- | --- | --- | --- | --- | --- |
| –/+ z-VAD-fmk | – | – | + | – | + | – | + | – | + | – | + |
| <2*n* (% of cells) | 9.8 ± 1.86 | 10.8 ± 3.00 | 7.2 ±1.20 | 15.0 ± 2.93 | 6.2 ± 0.13 | 25.3 ± 3.16 | 7.6 ± 0.13 | 31.3 ± 5.56 | 9.0 ± 0.51 | 55.6 ± 4.89 | 17.5 ± 0.87 |
| 2*n* (% of cells) | 43.5 ± 0.28 | 24.1 ± 4.53 | 22.3 ± 3.98 | 6.3 ± 1.89 | 6.1 ± 0.98 | 8.9 ± 3.71 | 3.3 ± 0.84 | 9.5 ± 3.76 | 1.9 ± 0.22 | 5.3 ± 0.58 | 1.3 ± 0.36 |
| *>*2*n*–*<*4*n* (% of cells) | 24.0 ± 0.38 | 27.5 ± 0.40 | 29.8 ± 0.44 | 12.9 ± 5.64 | 9.9 ± 2.71 | 14.3 ± 4.18 | 3.6 ± 0.53 | 19.4 ± 4.20 | 2.2 ± 0.31 | 10.6 ± 0.51 | 1.1 ± 0.24 |
| 4*n* (% of cells) | 15.9 ± 0.17 | 30.2 ± 4.18 | 33.0 ± 3.22 | 51.9 ± 5.87 | 57.6 ± 1.96 | 22.3 ± 6.51 | 31.8 ± 5.91 | 12.1 ± 0.76 | 18.2 ± 1.18 | 8.1 ± 0.58 | 16.4 ± 2.62 |
| *>*4*n* (% of cells) | 6.8 ± 1.66 | 7.3 ± 1.89 | 7.9 ± 1.96 | 13.9 ± 4.62 | 20.3 ± 5.04 | 29.2 ± 6.51 | 53.7 ± 7.38 | 27.8 ± 5.16 | 68.8 ± 1.84 | 20.3 ± 3.22 | 63.8 ± 3.24 |

Cells were exposed to 30 nM CFI-900495 for the indicated times; 20 µM z-VAD-fmk was applied 1 h before treatment with CFI-900495. The percentage of cells in each cell cycle phase was determined by flow-cytometric analysis of PI-stained ethanol-fixed cells. Means ± SEM of three separate measurements are shown.

**Table S8. Time course of centrinone-induced cell cycle effects in WE-68 cells.**

| Incubation time | 0 h | 12 h | | 24 h | | 36 h | | 48 h | | 72 h | |
| --- | --- | --- | --- | --- | --- | --- | --- | --- | --- | --- | --- |
| –/+ z-VAD-fmk | – | – | + | – | + | – | + | – | + | – | + |
| <2*n* (% of cells) | 11.2 ± 3.33 | 9.6 ± 2.82 | 8.4 ± 2.51 | 10.7 ± 3.11 | 5.9 ± 1.64 | 18.6 ± 5.18 | 9.3 ± 3.71 | 25.1 ± 6.07 | 14.7 ± 4.91 | 50.8 ± 3.33 | 24.8 ± 4.38 |
| 2*n* (% of cells) | 44.8 ± 3.01 | 39.6 ± 0.87 | 47.2 ± 6.44 | 45.6 ± 3.33 | 43.8 ± 3.38 | 30.8 ± 2.87 | 35.1 ± 4.82 | 21.7 ± 3.98 | 24.2 ± 4.89 | 10.2 ± 3.13 | 13.6 ± 4.24 |
| *>*2*n*–*<*4*n* (% of cells) | 20.8 ± 2.67 | 21.5 ± 1.78 | 19.1 ± 5.60 | 17.3 ± 5.00 | 18.6 ± 5.58 | 18.5 ± 2.76 | 17.9 ± 3.51 | 15.2 ± 0.87 | 14.0 ± 2.02 | 11.9 ± 2.09 | 11.5 ± 1.62 |
| 4*n* (% of cells) | 16.7 ± 0.92 | 23.6 ± 2.84 | 19.5 ± 4.27 | 20.3 ± 1.24 | 24.5 ± 3.93 | 25.3 ± 1.80 | 29.1 ± 3.91 | 31.0 ± 2.09 | 34.1 ± 0.56 | 19.5 ± 2.64 | 35.9 ± 1.82 |
| *>*4*n* (% of cells) | 6.5 ± 1.49 | 5.7 ± 1.87 | 5.8 ± 1.16 | 6.0 ± 1.24 | 7.2 ± 2.16 | 6.8 ± 1.73 | 8.5 ± 1.73 | 7.0 ± 1.27 | 12.9 ± 2.56 | 7.7 ± 2.09 | 14.2 ± 2.40 |

Cells were exposed to 2 µM centrinone for the indicated times; 20 µM z-VAD-fmk was applied 1 h before treatment with centrinone. The percentage of cells in each cell cycle phase was determined by flow-cytometric analysis of PI-stained ethanol-fixed cells. Means ± SEM of three separate measurements are shown.

**Supplementary Figures**

**Fig. S1** Relative *PLK4* expression in Ewing’s sarcoma cell lines. *PLK4* mRNA levels were determined by real-time RT-PCR and normalised to *β-2-microglobulin* mRNA levels. *PLK4* expression levels in Ewing’s sarcoma cells are shown as percentage of the *PLK4* expression level in HeLa cells. Means ± SEM of two separate measurements are shown.

**Fig. S2** Antineoplastic effects of PLK4i in combination with etoposide in Ewing's sarcoma cells. Cells were exposed to CFI-400945 or centrinone in combination with etoposide for 48 h. Cell viability was determined by resazurin assay. Means ± SEM of each three separate measurements are shown.

CFI-400945

Centrinone

WE-68

SK-ES-1

A673


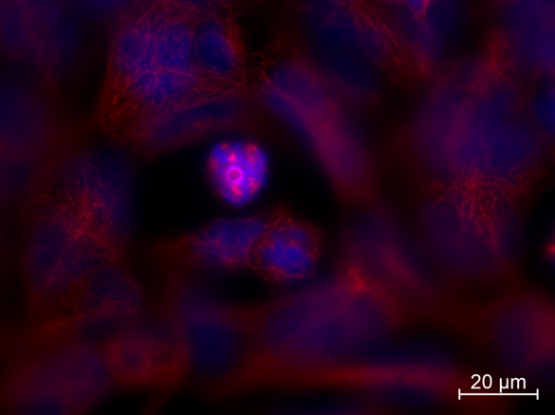

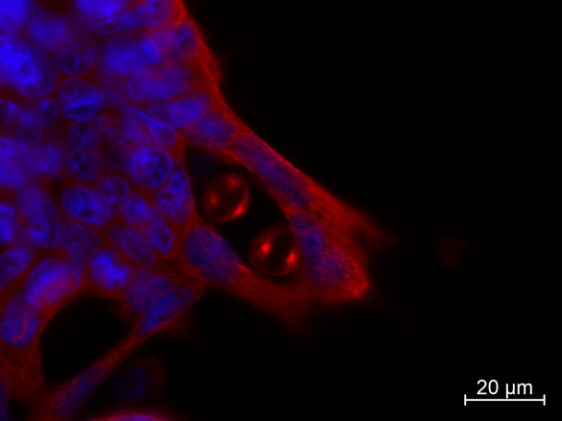

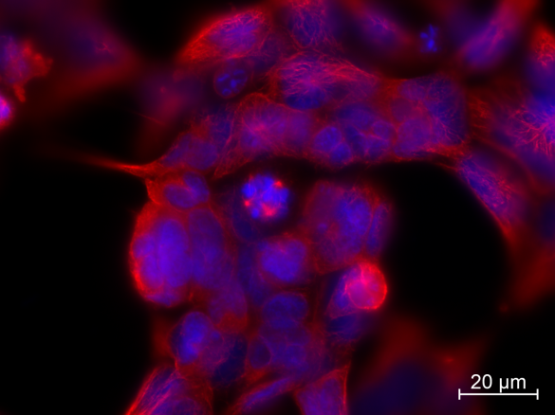

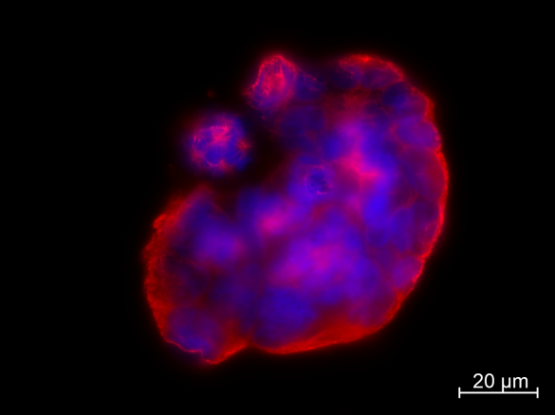

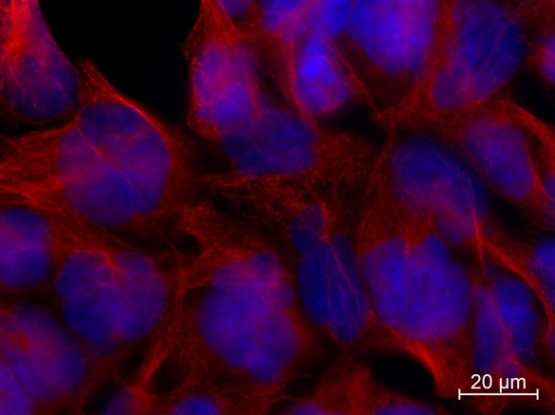

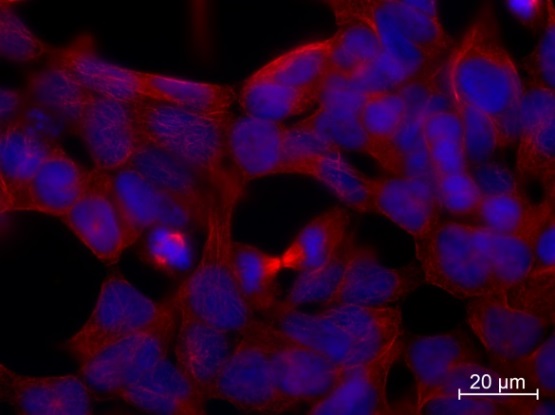

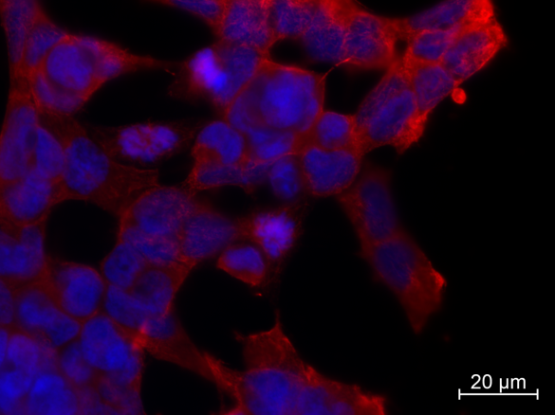

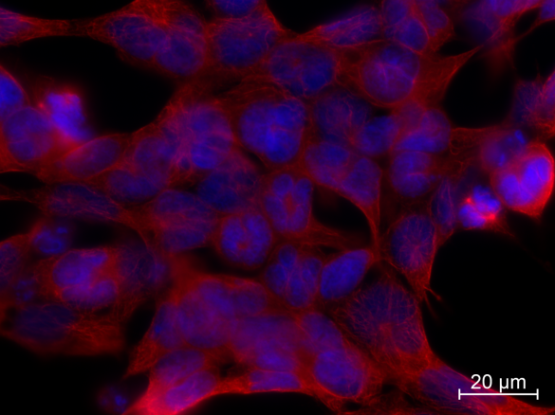


**Fig. S3** CFI-400945 induces multipolar spindles in Ewing‘s sarcoma cells. **a, b** WE-68 cells were exposed to CFI-400945 for 24 h; **b** z-VAD-fmk was applied 1 h before treatment with CFI-400945. The blue signal shows DAPI staining and the red signal shows *α*-tubulin staining. Images are representative of three independent experiments.

Control

3-pole

spindle

15 nM CFI-400945

4-pole

spindle

Multinuclear

cells

Multipolar

spindle

**b**

**a**

15 nM CFI-400945

10 nM CFI-400945

Control

20 nM CFI-400945

20 nM CFI-400945

15 nM CFI-400945

Multipolar

spindle

Multinuclear

cells

**+ 20 µM z-VAD-fmk**
